# Case for diagnosis. Erythematous and infiltrated plaques in the infrahyoid region^☆^^☆☆^

**DOI:** 10.1016/j.abd.2020.03.022

**Published:** 2020-11-20

**Authors:** Natália Tenório Cavalcante Bezerra, Antonio Pedro Mendes Schettini, André Luiz Leturiondo, Liana Hortencia Miranda Tubilla Mathias

**Affiliations:** Fundação Alfredo da Matta, Manaus, AM, Brazil

**Keywords:** Diagnosis, differential, HIV infections, Leprosy

## Abstract

Leprosy is a chronic infectious disease caused by *Mycobacterium leprae* and, depending on the host immune status, presents different clinical forms. This report describes the case of a 46-year-old man who had hypoesthetic lesions in the infrahyoid region for 30 days. The bacilloscopy was negative. The anatomopathological examination showed alterations corresponding to the tuberculoid pole (epithelioid histiocytes) and virchowian pole (foamy histiocytes), compatible with borderline-virchowian leprosy (Ridley and Jopling classification). Rapid tests for HIV I, II, and syphilis were positive, with a CD4 count of 223. The patient started treatment with multibacillary multidrug therapy, antiretroviral therapy, and benzathine penicillin, with marked clinical improvement in two months.

## Case report

**Figure 1:**
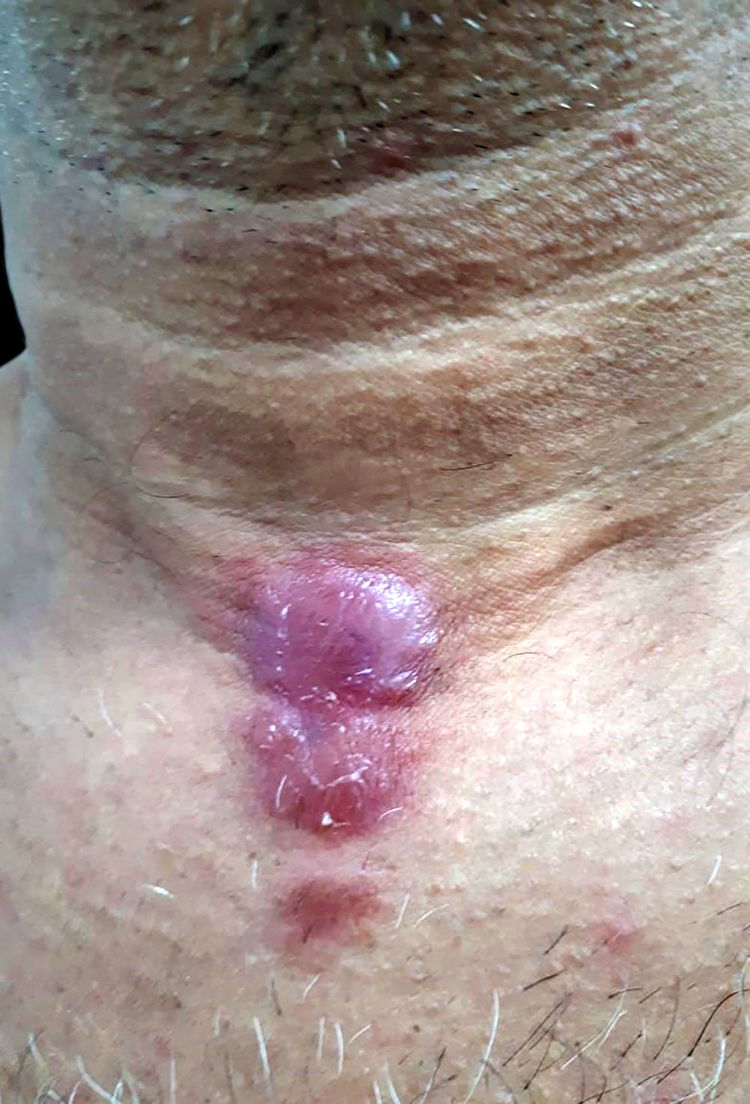
Erythematous and infiltrated plaques, confluent in the infrahyoid region, with well-defined edges.

**Figure 2:**
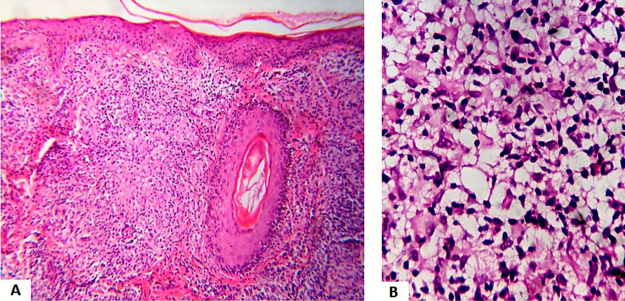
(A), Dermal granulomatous inflammatory process with epithelioid cells, giant cells and lymphocytes (Hematoxylin & eosin, ×100). (B), Dermal inflammatory process with foamy histiocytes (Hematoxylin & eosin, ×400).

**Figure 3:**
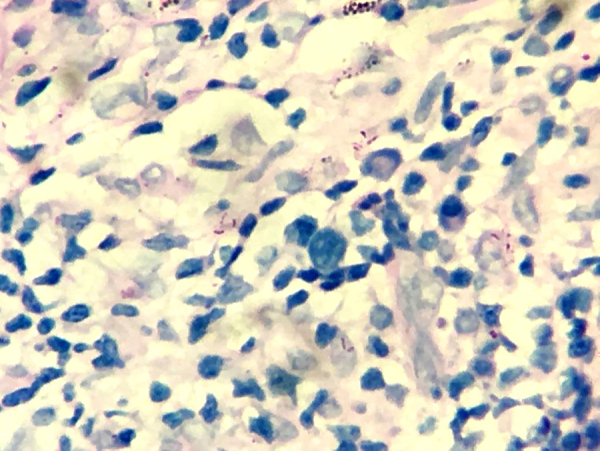
Presence of several acid-fast bacilli in the dermis (Wade, ×100).

**Figure 4:**
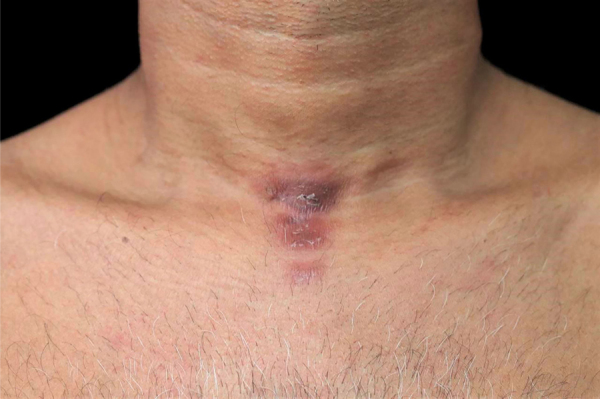
Aspect of the lesions after two months of multidrug treatment for multibacillary leprosy. The lesions are impregnated with clofazimine.

A 46-year-old male presented two erythematous infiltrated plaques for 30 days, with changes in thermal, tactile and painful sensitivity on the infrahyoid region (Fig. 1). He also reported diarrhea and the loss of 8 kg in four months. The bacilloscopy was negative. Histopathology showed a diffuse inflammatory dermal infiltrate made up of epithelioid histiocytes and focal foam cells, lymphocytes, and giant Langhans cells, distributed around vessels, adnexa, and nerves (Fig. 2). Several isolated and fragmented bacilli were demonstrated in the papillary dermis using the Wade-Fite method (Fig. 3). The polymerase chain reaction (PCR) was positive for Mycobacterium leprae, and culture for other mycobacteria was negative. Serology for syphilis and HIV was positive, with a CD4 of 223 and a viral load of 221,601 copies. With the diagnosis of borderline-virchowian leprosy, he started treatment with multibacillary multidrug therapy, benzathine penicillin, and antiretroviral therapy, with partial regression of the lesions after two months (Fig. 4).

## What is your diagnosis?

a) Atypical mycobacteriosis
b) Sarcoidosis
c) Leprosy
d) Cutaneous tuberculosis

## Discussion

Due to the interactions between Mycobacterium leprae (*M. leprae*) and the host's immune response, leprosy presents great clinical polymorphism, justifying the several classifications of the disease proposed throughout history.^1^

There was an assumption that, in patients co-infected with leprosy and HIV, the compromised immune system could interfere in several clinical-pathological aspects of leprosy.^2^ However, studies have shown that there is no direct impact on the rate of detection of the disease in HIV-positive patients, and that the classic clinical forms prevail. Leprosy reactions also do not appear to be more frequent, and the treatment is effective at the usual doses and duration.^3^

In the reported case, the patient had an unusual clinical picture, mimicking various diseases, making it difficult to diagnose and prescribe the appropriate therapeutic regimen. The conclusion was established by tests not available in primary healthcare units. Bacilloscopy, performed by intradermal scraping of four sites, one of them being the infrahyoid lesion, was negative even after being repeated. In an unusual way, the histopathological examination showed that the bacilli were located at the limit between the papillary and reticular dermis and were restricted to a certain field, rather than distributed diffusely. The clinical-pathological categorization of the patient then became a challenge.

The authors chose to classify it as borderline-virchowian (Ridley and Jopling classification) based on the histopathological findings that showed characteristics of the tuberculoid pole (epithelial granulomas) and the virchowian pole (vacuolated histiocytes) in the same biopsy, possibly registering a transition from the borderline-borderline form to borderline-virchowian.^4^, ^5^

Routine examinations at the reference service enabled the diagnosis of comorbidities (AIDS and syphilis) in this patient. It was also possible to perform tests such as histopathology and PCR, which allowed the diagnostic conclusion of leprosy in an atypical clinical lesion.

It is important to emphasize that the basic health care network must have reference services which provide technical capabilities to confirm or rule out the diagnosis of leprosy in patients whose clinical presentation differs from the usual.

## Financial support

None declared.

## Authors’ contributions

Natália Tenório Cavalcante Bezerra: Drafting and editing of the manuscript; critical review of the literature.

Antonio Pedro Mendes Schettini: Approval of the final version of the manuscript; drafting and editing of the manuscript; critical review of the manuscript.

André Luiz Leturiondo: Intellectual participation in propaedeutic and/or therapeutic conduct of studied cases.

Liana Hortência Miranda Tubilla Mathias: Intellectual participation in propaedeutic and/or therapeutic conduct of studied cases.

## Conflicts of interest

None declared.
